# *SDHB* exon 1 deletion: A recurrent germline mutation in Colombian patients with pheochromocytomas and paragangliomas

**DOI:** 10.3389/fgene.2022.999329

**Published:** 2023-01-04

**Authors:** María Carolina Manotas, Ana Lucía Rivera, Ana Milena Gómez, Patricia Abisambra, Gonzalo Guevara, Vilma Medina, Sandra Tapiero, Antonio Huertas, Julián Riaño-Moreno, Juan Carlos Mejía, Angélica María Gonzalez-Clavijo, Mireya Tapiero-García, Andrés Arturo Cuéllar-Cuéllar, Luis Felipe Fierro-Maya, María Carolina Sanabria-Salas

**Affiliations:** ^1^ Medical Subdirection, Instituto Nacional de Cancerología, Bogotá, Colombia; ^2^ Department of Medicine, Universidad del Norte, Barranquilla, Colombia; ^3^ Subdirection of Research, Instituto Nacional de Cancerología, Bogotá, Colombia

**Keywords:** pheochromocytomas, paragangliomas, germline mutation, succinate dehydrogenase subunit B, human, neoplastic syndromes, hereditary

## Abstract

Pheochromocytomas (PCCs) and paragangliomas (PGLs) (known as PPGL in combination) are rare neuroendocrine tumors of the adrenal medulla and extra-adrenal ganglia. About 40% of the patients with PPGL have a hereditary predisposition. Here we present a case-series of 19 unrelated Colombian patients with a clinical diagnosis of PPGL tumors that underwent germline genetic testing as part of the Hereditary Cancer Program developed at the Instituto Nacional de Cancerología, Colombia (INC-C), the largest reference cancer center in the country. Ten of 19 patients (52.63%) were identified as carriers of a pathogenic/likely pathogenic (P/LP) germline variant in a known susceptibility gene. The majority of the P/LP variants were in the *SDHB* gene (9/10): one corresponded to a nonsense variant c.268C>T (p.Arg90*) and eight cases were found to be carriers of a recurrent CNV consisting of a large deletion of one copy of exon 1, explaining 42% (8/19) of all the affected cases. Only one additional case was found to be a carrier of a missense mutation in the *VHL* gene: c.355T>C (p.Phe119Leu). Our study highlights the major role of *SDHB* in Colombian patients with a clinical diagnosis of PGL/PCC tumors and supports the recommendation of including the analysis of large deletions/duplications of the *SDHB* gene as part of the genetic counselling to improve the detection rate of hereditary cases and their clinical care.

## 1 Introduction

Pheochromocytoma (PCC) was first described by Fränkel in 1886; since then, important discoveries and advances have been made in relation to diagnosis, genetics, and management (Wiseman et al., 2019). PCC is a rare neuroendocrine tumor, which originates from the chromaffin cells of the adrenal medulla, and is related to paraganglioma (PGL), which originates from extra-adrenal chromaffin cells (Aygun and Uludag, 2020); both are histologically identical (Berends et al., 2018). Chromaffin cells of the adrenal medulla are major components of the autonomic nervous system that releases catecholamines into the blood stream; interestingly, recent studies have uncovered new cellular origin of chromaffin cells from Schwann cell precursors, and understanding these differentiation pathways may help to comprehend the origin of these tumors and improve their clinical management (Furlan et al., 2017; Kastriti et al., 2019). Most PCCs and PGLs are secreting or functioning endocrine tumors, and their clinical presentation is associated with excessive secretion of catecholamines (epinephrine, norepinephrine, and dopamine) and their metabolites (Aygun and Uludag, 2020). PCCs and PGLs, in combination, are called PPGL (Boyd et al., 2019). Sympathetic PGLs cause excess catecholamines, while parasympathetic PGLs are mostly non-secreting. Symptoms of PCC/PGL are due to mass effects or catecholamine hypersecretion (e.g., sustained or paroxysmal elevations in blood pressure, headache, profuse sweating, intense palpitations, paleness, and apprehension or anxiety) (Else et al., 2018). Hypertension is present in 80–90% of the patients, while approximately 25% of the patients present with the classically defined symptom triad of headache, sweating, and palpitations (Benn et al., 2015; Aygun and Uludag, 2020). Diagnosis is typically confirmed by determination of plasma and urinary fractionated catecholamines and metanephrines, being the determination of plasma free metanephrines the test of choice (Lenders et al., 2002). Non-hormone-producing PGLs are found in the head and neck area, and less frequently in the thorax; symptoms related to this type of tumors are due to pressure on surrounding nerves and include hearing loss, pulsatile tinnitus, cough, hoarseness, dysphagia, facial paralysis, pain or abnormal tongue motility (Benn et al., 2015). Tumor location is determined based on imaging studies, such as magnetic resonance imaging, computed tomography, positron-emission tomography, and/or scintigraphy (Oleaga and Goñi, 2008).

PPGLs have an incidence between 2 and 8/1,000,000 inhabitants; they occur most frequently between 40 and 50 years and mostly in women (55%) (Farrugia et al., 2017; Garcia-Carbonero et al., 2021). However, 20% of the cases are pediatric with a peak of presentation between 11 and 13 years of age (Aygun and Uludag, 2020; Jain et al., 2020). At the Instituto Nacional de Cancerología, Colombia (INC-C), in 2019, three new cases of malignant PCC and one case of malignant PGL with unknown primary location were reported (Anuario estadístico 2019, 2021). In 2020 COVID-19 affected, the number of new cases was similar with three new cases of malignant PCC and two cases of malignant PGL with unknown primary location (Anuario estadístico 2020, 2021). The proportion of women was 4/5 in 2020, while in 2019 the proportion of women was 2/4.

PPGLs can occur sporadically or as part of different hereditary tumor syndromes (Welander et al., 2011; Fishbein, 2016; Aygun and Uludag, 2020). Several studies have reported that 30–40% of the cases are caused by germline mutations (Neumann et al., 2002; Welander et al., 2011; Gimenez-Roqueplo et al., 2012; Dahia, 2014; Favier et al., 2015; Fishbein, 2016; Aygun and Uludag, 2020). The syndromes associated with inherited PGL/PCC are characterized by a predisposition to the development of PGLs distributed along the paravertebral axis from the base of the skull to the pelvis, as well as PCCs that are confined to the adrenal medulla (Else et al., 2018). Therefore, the diagnosis of a hereditary PGL/PCC syndrome should be suspected in any individual with a diagnosis of PGL or PCC, and the current recommendation is to offer germline genetic studies to all of these patients, regardless of family history or age at presentation (Plouin et al., 2016; Aygun and Uludag, 2020), especially if the presentation is multiple, multifocal, recurrent, or early-onset (<35 years) (Muth et al., 2012). About 15–20% of the hereditary cases are associated with pathogenic/likely pathogenic (P/LP) germline variants in genes that encode different subunits of the succinate dehydrogenase complex-SDH (*SDHA, SDHB, SDHC*, and *SDHD*, and the cofactor *SDHAF2*) (Else et al., 2018; Garcia-Carbonero et al., 2021). Also, 9% of the cases are associated with Von Hippel-Lindau syndrome - VHL (*VHL* gene), 5% corresponds to multiple endocrine neoplasia 2 syndrome—MEN2 (*RET* proto-oncogene), and 2% are associated with neurofibromatosis type 1 syndrome—NF1 (*NF1* gene). Other genes associated with hereditary PGL/PCC are *TMEM127, MAX, FH, MEN1, EGLN2, MDH2, SLC25A11*, and *DLST*, with lower frequencies (<1–2%) (Dahia, 2014; Garcia-Carbonero et al., 2021).

In this study, we present for the first time the clinical characterization and spectrum of causative gene variants identified in a series of unrelated Colombian patients affected with PCC and/or PGL.

## 2 Materials and methods

### 2.1 Type of study and patients

This is a registry-based study on patients affected with PPGL tumors that were included in the Hereditary Cancer Program developed in the INC-C. This institutional Program was approved by the Scientific Committee of the INC-C in 2017 and was established to offer germline genetic testing to cancer patients at the INC-C as a care service for their comprehensive management. Patients participating in this institutional Program accepted to be donors at the tissue Biobank from the INC-C named “Banco Nacional de Tumores Terry Fox” and signed an informed consent for DNA biobanking and future research studies. The Program’s registry is being implemented for epidemiological reports. A total of 20 unrelated patients affected with PGL or PCC were identified in the registry from April 2018 to December 2021. To confirm a hereditary PGL/PCC syndrome, most of them were referred from the clinical oncology or endocrinology services at the INC-C to the Institutional´s genetic counselling service, and only five of them were managed by a medical geneticist at external institutions. All patients were offered genetic testing for diagnostic purposes, given the high frequency of germline mutations reported for these patients and international recommendations. Only 1 out of the 20 patients did not accept the genetic study. Of the 19 patients with genetic results, 14 were performed at the INC-C and 5 corresponded to studies carried out in external laboratories.

### 2.2 DNA extraction, library preparation, and massive sequencing (NGS)

Germline DNA was extracted from peripheral blood using the Quick-DNA TM Miniprep Plus Kit (Zymo Research, United States) following the manufacturer’s instructions. DNA was quantified with the Qubit dsDNA BR kit (Invitrogen) and the NanoDrop™ 2000 (Thermo Scientific) equipment, and DNA quality was assessed with the Bioanalyzer High Sensitivity DNA Analysis Kit (Agilent). Library preparation and sequencing was performed as recommended by the manufacturer’s protocol for the Nextera Flex for Enrichment Illumina kit and the Canadian Consortia Inherited Cancer a customized probe panel (reference # 20011891; Illumina Inc., San Diego, United States), that targets 105 genes known to be strongly associated with inherited cancers—or candidate genes—and detects single nucleotide variants (SNVs) and small insertions and deletions (INDELs) (Table 1). Additionally, the multigene panel design allows inferring possible copy number variants (CNVs) in all genes with bioinformatics methods by using sequencing data. Briefly, the DNA sample was enriched for the target regions using the protocol by Nextera Flex for Enrichment (Illumina Inc., San Diego, CA) based on hybridization, and the libraries were sequenced using paired-end technology (2 × 251 cycles) in a MiSeq instrument (Illumina Inc., San Diego, CA). Unless otherwise indicated, all target regions were sequenced to a depth greater than 100X (if an allelic depth greater than 50X was not achieved, complementary analyses of the region of interest were performed by orthogonal methods). This assay focuses on the coding sequences of the genes included. Promoter regions, non-transcribed regions, and other non-coding regions are not included. NGS analysis was previously standardized and validated in our laboratories to sequence 12 samples simultaneously and according to the Analytical Performance results we obtained a sensitivity of 99.89% and a specificity of 99.99% for the accurate detection of SNVs and small INDELs genetic variants using the commercial bioinformatics software developed by Sophia Genetics (Saint-Sulpice, Switzerland).

**TABLE 1:** Genes included in the multigene panel.

*AIP*	*ALK*	*APC*	*ATM*	*BAP1*	*BARD1*	*BLM*	*BMPR1A*	*BRCA1*	*BRCA2*	*BRIP1*
*BUB1B*	*CASR*	*CDC73*	*CDH1*	*CDK4*	*CDKN1B*	*CDKN1C*	*CDKN2A*	*CEBPA*	*CEP57*	*CHEK2*
*CYLD*	*DDB2*	*DICER1*	*DIS3L2*	*EGFR*	*EPCAM*	*ERCC2*	*ERCC3*	*ERCC4*	*ERCC5*	*EXT1*
*EXT2*	*EZH2*	*FANCA*	*FANCB*	*FANCC*	*FANCD2*	*FANCE*	*FANCF*	*FANCG*	*FANCI*	*FANCL*
*FANCM*	***FH***	*FLCN*	*GATA2*	*GNAS*	*GPC3*	*HNF1A*	*HRAS*	*KIT*	***MAX***	***MEN1***
*MET*	*MLH1*	*MRE11A*	*MSH2*	*MSH6*	*MUTYH*	*NBN*	***NF1***	*NF2*	*NSD1*	*PALB2*
*PDE4D*	*PHOX2B*	*PMS1*	*PMS2*	*POLD1*	*POLE*	*PPM1D*	*PRF1*	*PRKAR1A*	*PTCH1*	*PTEN*
*RAD50*	*RAD51C*	*RAD51D*	*RB1*	*RECQL4*	***RET***	*RHBDF2*	*RUNX1*	*SBDS*	***SDHA***	***SDHAF2***
***SDHB***	***SDHC***	***SDHD***	*SLX4*	*SMAD4*	*SMARCB1*	*STK11*	*SUFU*	***TMEM127***	*TP53*	*TSC1*
*TSC2*	***VHL***	*WRN*	*WT1*	*XPA*	*XPC*

Genes with the best evidence of their association with hereditary PGL/PCC, are in *bold* (Fishbein and Nathanson, 2012; Dahia, 2014).

### 2.3 Variant calling, interpretation of genetic data, and mutation detection

Sequence reads in FastQC files were aligned to the hg19 human reference genome with the Burrows-Wheeler Aligner (BWA) tool. Variant calling and annotation of SNVs and INDELs were carried out with the SOPHiA DDM^®^ platform using the ILL1IC1G3_TSC algorithm (Sophia Genetics, Saint-Sulpice, Switzerland). This algorithm also allows inferring CNVs from sequence data for all genes included in the panel, and the detection of ALU elements. The genetic variants detected by the SOPHiA algorithm were reviewed by an oncogeneticist and a biologist trained in genetics for interpretation and delivery of genetic results at the post-test consultation. The Human Genome Variation Society (HGVS) nomenclature (http://www.hgvs.org/) was used in the genetic report, and the five-tier criteria of the American College of Medical Genetics and Genomics (ACMG) for variant classification were implemented for variant classification (Richards et al., 2015). The genetic variants reported in this manuscript were included in an institutional automated or manual curation exercise, to update their classification.

### 2.4 Large deletions/duplication analyses

The gold-standard method to identify large genomic deletions and duplications is Multiplex Ligation-dependent Probe Amplification (MLPA). Among the eight patients describe here as carriers of the exon 1 deletion of the *SDHB* gene, two corresponded to cases with external results that included both NGS and MLPA analysis, and these cases were included in the Hereditary Cancer Program through the tissue Biobank as positive controls for future standardization purposes. The other six, corresponded to cases in which a CNV in the *SDHB* was detected through NGS at our genetic laboratory. All of these cases were also analyzed with MLPA at an external genetic laboratory. Positive control samples from our tissue Biobank were provided for this step.

Briefly, the SALSA MLPA Probemix P226 SDH was used for confirmation purposes. This consists in a semi-quantitative assay for both an *in vitro* diagnostic and research use. Regions covered with this assay are: *SDHD 11q23.1; SDHB 1p36.1; SDHC 1q23.3; SDHAF1 19q13.12; SDHAF2 11q12.2* (https://www.mrcholland.com/product/P226/2401?). Detection by fragment analysis was performed following the manufacturer’s instructions (MRC-Holland, Amsterdam, Netherlands): 1) DNA extraction and denaturation; 2) Hybridization with the specific probes; 3) Ligation and amplification by PCR reaction; 4) Capillary electrophoresis of the amplified products in the ABI 3500 Genetic Analyzer sequencer (Applied Biosystems); and 5) Analysis of results with the program bioinformatician Coffalyzer.

### 2.5 Statistical analysis

The diagnosis of a hereditary PGL/PCC syndrome was established with the identification of a germline P/LP variant in the affected patient, in a gene known to be associated with increased susceptibility to developing PGL or PCC tumors, such as: *MAX, NF1, RET, SDHA, SDHAF2, SDHB, SDHC, SDHD, TMEM127, VHL, FH* and *MEN1* (Table 1). Demographic data (sex, age at diagnosis), tumor type, clinical presentation, and results of the molecular study, were analyzed. Data were summarized using descriptive statistics.

## 3 Results

### 3.1 Demographic and clinical characteristics of patients

Of the 19 patients with genetic results, the majority were women (n = 12, 63.16%) and the mean age at first presentation was 34.8 years (range 9–60 years). Three patients (15.79%) had PGL or PCC of pediatric presentation (<18 years). Overall, 68.42% (13/19) had catecholamine-secreting tumors (or their metabolites), and 47.37% (9/19) had metastatic tumors (Table 2).

**TABLE 2:** Clinical characteristics and genetic test results of patients with pheochromocytoma/paraganglioma (n = 19).

Patient	Sex	Age (years at diagnosis)	Tumors	Tumor location	Active (catecholamines or metabolites)	Metastasis (site)	Genetic results			Type	Variant classification	Diagnosed syndrome (AD/AR)
							Gene	Variant	Zygosity			
1	F	20	PGL	Retroperitoneum (celiac trunk)	Yes (normetanephrine)	No	*SDHB*	Exon 1 deletion	Het	CNV	P	Hereditary PGL/PCC (AD)
2	F	26	PGL	Unilateral carotid body/retroperitoneum*	Yes (norepinephrine)	No	*SDHB*	Exon 1 deletion	Het	CNV	P	Hereditary PGL/PCC (AD)
3	F	42	PCC	Unilateral adrenal (local relapse)	No	No	*SDHB*	Exon 1 deletion	Het	CNV	P	Hereditary PGL/PCC (AD)
4	M	42	PGL	Extra adrenal*/Unilateral carotid body	Yes (norepinephrine)	Yes (bone, lung)	*SDHB*	Exon 1 deletion	Het	CNV	P	Hereditary PGL/PCC (AD)
5	M	47	PGL	Cervical paravertebral spine	Yes (normetanephrine)	Yes (bone, liver)	*SDHB*	Exon 1 deletion	Het	CNV	P	Hereditary PGL/PCC (AD)
6	M	40	PCC	Unilateral adrenal	Yes (normetanephrine)	Yes (bone, liver, lymph nodes)	*SDHB*	Exon 1 deletion	Het	CNV	P	Hereditary PGL/PCC (AD)
7	M	37	PGL	Unknown primary	Yes (norepinephrine, dopamine)	Yes (bone, lymph nodes)	*SDHB*	Exon 1 deletion	Het	CNV	P	Hereditary PGL/PCC (AD)
8	F	13	PGL	Lumbosacral	Yes (metanephrine, normetanephrine)	No	*SDHB*	Exon 1 deletion	Het	CNV	P	Hereditary PGL/PCC (AD)
9	M	54	PGL	Retroperitoneum (in front of aorta and cava)	Yes (epinephrine, norepinephrine, metanephrine)	Yes (bone, lymph nodes)	*SDHB*	c.268C>T (p.Arg90*)	Het	Nonsense	P	Hereditary PGL/PCC (AD)
10	M	9	PCC	Unilateral adrenal	Yes (normetanephrine)	No	*VHL*	c.355T>C (p.Phe119Leu)	Het	Missense	P	Von Hippel-Lindau (AD)
11	F	31	PCC	Unilateral adrenal	Yes (norepinephrine)	No	*RAD51D*	c.94_95del (p.Val32Phefs*67)	Het	Frameshift	P	Breast-ovarian cancer, familial, susceptibility to, 4 (AD)
12	F	26	PGL	Vagus nerve, hypoglossal nerve, and right vocal cord (locoregional progression)	No	No	*ATM*	c.610G>A (p.Gly204Arg)	Het	Missense	VUS	N/A
13	F	60	PCC	Unilateral adrenal	No	Yes (bone, liver)	*MEN1*	c.541G>A (p.Ala181Thr)	Het	Missense	VUS	N/A
14	F	53	PGL	Unilateral carotid body	No	No	*RECQL4*	c.1040G>A (p.Arg347His)	Het	Missense	VUS	N/A
15	F	44	PCC	Unilateral adrenal	Yes (metanephrine, normetanephrine)	No	variant not detected		N/A	N/A	N/A	N/A
16	M	29	PGL	Retroperitoneum (paraaortic)	Yes (metanephrine, normetanephrine)	Yes (bone, liver, lymph nodes)	variant not detected		N/A	N/A	N/A	N/A
17	F	15	PGL/PCC	Bilateral adrenal*/Unilateral carotid body/Paraaortic* (PASS >6)	Yes (metanephrine, normetanephrine, Dopamine)	No	variant not detected		N/A	N/A	N/A	N/A
18	F	32	PGL	Unilateral carotid body	No	Yes (bone, liver, mesentery)	variant not detected		N/A	N/A	N/A	N/A
19	F	42	PGL	Unilateral carotid body	No	Yes (bone, liver)	variant not detected		N/A	N/A	N/A	N/A

*PCC:* pheochromocytoma, *PGL:* paraganglioma, *Het:* heterozygous, *CNV:* copy number variation*,* P: pathogenic, *LP:* likely pathogenic, *VUS:* variant of uncertain significance, *N/A:* not applicable, *PASS:* pheochromocytoma of the adrenal gland scaled score, *AD:* autosomal dominant, *AR:* autosomal recessive, * indicates active tumor.

Regarding tumor location and multifocality or multiple presentation, five patients (26.32%) presented PGL of the head and neck, three patients (38.5%) developed extra-adrenal abdominal PGL with involvement of the retroperitoneal region, and one patient (5.2%) had a PGL located at the lumbosacral level. Multiple PCC/PGL tumors were found in 15.79% (3/19) of the patients; two of them with multiple primary PGLs located in the head and neck and abdomen (retroperitoneum), and the third one developed bilateral PCC with various PGLs tumors located in the head and neck and abdomen. Finally, six patients (31.5%) had unilateral PCC, while another patient (5.2%) presented with metastatic PGL of unknown primary origin (Table 2).

About 26.32% (5/19) of the patients also developed other primary tumors different than PPGL (Table 3). Family history of PGL or PCC tumors were reported by 15.79% (3/19) of the cases (Table 3), while another 11 patients (57.89%) reported relatives affected with different tumors (data not shown).

**TABLE 3:** Characteristics of adult patients with sporadic PCC/PGL and hereditary PGL/PCC associated with exon 1 deletion in the *SDHB* gene.

	Sporadic PCC/PGL^a^ (n = 8) (%)	Hereditary PGL/PCC associated with exon 1 deletion in the *SDHB* gene (n = 7) (%)
Sex		
Male	1 (12.5%)	4 (57.1%)
Female	7 (87.5%)	3 (42.9%)
Age at diagnosis		
Mean (range)	39.6 (26–60)	36.3 (20–47)
Tumor type		
PCC	3 (37.5%)	2 (28.6%)
PGL	5 (62.5%)	5 (71.4%)
Tumor characteristics		
Active	3 (37.5%)	6 (85.7%)
Multiple	0	2 (28.6%)
Metastatic	4 (50%)	4 (57.1%)
Cases with additional non-PCC/PGL primary tumors	2 (25%)	3 (42.9%)
Family members with PCC/PGL	0	3 (42.9%)
Family members with non-PCC/PGL tumors	6 (75%)	3 (42.9%)

*PCC:* pheochromocytoma, *PGL:* paraganglioma.

^a^ Sporadic PCC/PGL, cases include those with negative genetic testing result, those with only VUS, and the sporadic PCC found to be a carrier of a P/LP variant in *RAD51D* (not associated with hereditary PGL/PCC syndrome).

### 3.2 Germline genetic results and observed phenotypes

**FIGURE 1:**
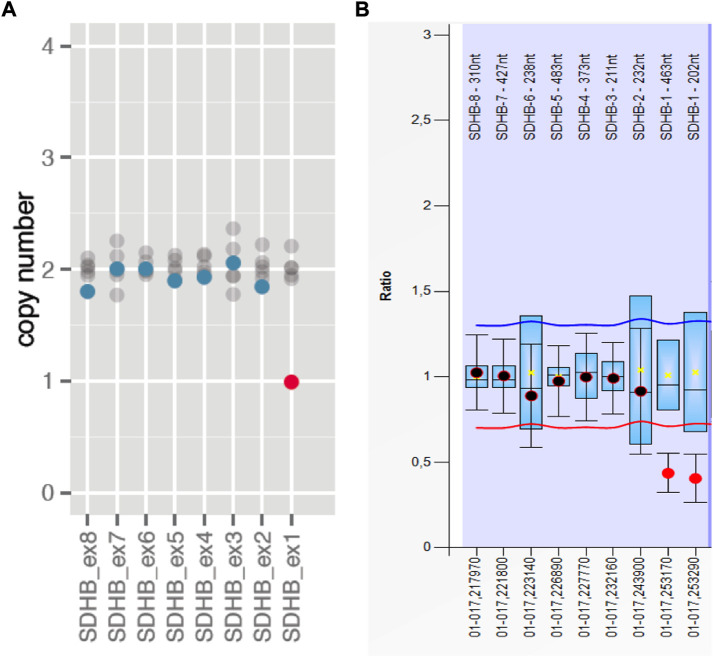
Recurrent CNV in the *SDHB* gene in Colombians consisting in a large deletion of one copy of exon 1. **(A)** The graphic shows the copy number of the carrier (dots in color blue/red) and if it is similar (blue dots) or different (red dots) compared to the median of the copy numbers of each exon (1–8) among 8 different samples (gray dots) included in the same run. Graphic generated and extracted from SOPHIA Genetics. **(B)** Corresponding graphic obtained with MLPA confirmatory assay.

A heterozygous germline P/LP variant in a known PPGL susceptibility gene was detected in 52.63% (10/19) of the Colombian patients. In nine cases the affected gene was *SDHB* and were diagnosed with hereditary PGL/PCC syndrome (MedGen UID: 313270). Only one case was found to be a carrier of a disease-causing variant in the *VHL* gene and was diagnosed with VHL syndrome (MedGen UID: 42458) (Table 2). It is noteworthy that among the nine cases with a *SDHB* mutation, only one corresponded to a nonsense variant *SDHB*: c.268C>T (p.Arg90*), while the remaining eight are carriers of a recurrent CNV consisting of a large deletion of one copy of exon 1, explaining 80% (8/10) of the hereditary PGL/PCC cases and 42% (8/19) of all the cases affected with PGL or PCC tumors (Table 2; Figure 1). The patient with the nonsense variant in the *SDHB* gene was diagnosed at age 54 years with a metastatic secreting-PGL tumor, with neither a personal history of second primary tumors nor a family history of PGL/PCC tumors (Table 2). On the other hand, the patient with the missense mutation in the *VHL* gene: c.355T>C (p.Phe119Leu) corresponds to a child diagnosed at 9 years with non-metastatic secreting-PCC tumor, not associated with second primaries or positive family history (Table 2).

An additional patient with an incidental finding of a unilateral, non-metastatic, secreting PCC tumor at age 31 years, carries a pathogenic variant in *RAD51D* gene that confers an autosomal dominant predisposition to breast and ovarian cancer (MedGen UID: 481975), but is not considered to be associated with a hereditary PGL/PCC syndrome (Table 2). In accordance with the above, 57.89% (11/19) of the cases are carriers of a P/LP germline variant associated with any hereditary cancer syndrome, 15.79% (n = 3) of the patients had a variant of uncertain significance (VUS), and 26.32% (n = 5) of the cases had negative results.

Table 3 describes some characteristics among patients >18 years with sporadic PGL or PCC (n = 8; these are cases with negative results or a VUS and also includes the carrier of the pathogenic variant in *RAD51D* since the PPGL tumor is considered unrelated) and hereditary cases with the recurrent CNV in the *SDHB* gene (n = 7). The mean age at diagnosis of the sporadic cases was 39.62 years; excluding the sporadic case with *RAD51D* mutation, the mean age at diagnosis would be 40.85 years in the sporadic group. On the other hand, the mean age at diagnosis in the hereditary PGL/PCC group with the recurrent CNV in *SDHB* was 36.29 years. In both groups, a higher proportion of PGL tumors was observed (62.5% and 71.4%, respectively), with lower frequency of PCC tumors (37.5% and 28.6%, respectively).

Other characteristics to mention in the group of hereditary cases is that 85.7% had secreting-tumors, 28.6% had multiple tumors, and 57.1% were metastatic at diagnosis. In patients with sporadic PGL or PCC, the proportion of secreting-tumors was only 37.5%, none of them had multifocal disease or multiple tumors, and 50% had metastatic disease (Table 3). Also, in the group of hereditary PGL/PCC more cases developed other primary tumors different from PPGL compared to the sporadic group (42.9% vs. 25%, respectively). Finally, none of the sporadic cases reported a family history of PGL/PCC tumors, although 75% of them had a positive family history for other tumors types; while in cases with the CNV in *SDHB*, 42.9% reported a positive family history of PPGL tumors and another 42.9% had a positive family history for other tumor types (Table 3). As for the age at diagnosis of metastatic disease, the mean in both groups was 44.25 years (data not shown).

Regarding the three patients with pediatric presentation (not included in Table 3), in two of them with NGS and MLPA studies, a P/LP variant associated with hereditary PGL/PCC was detected (Table 2). These are Patient 8 diagnosed at age 13 years with non-metastatic secreting-PGL tumor of lumbosacral location in whom the recurrent CNV in *SDHB* was identified, and Patient 10 diagnosed at age 9 years with non-metastatic secreting-PCC tumor, who carries the pathogenic variant in the *VHL* gene. The third pediatric patient corresponds to a case with a 14-gene NGS study reported as negative (without MLPA studies according to the EMR), but with a high suspicion of a hereditary PGL/PCC syndrome since the age at diagnosis was 15 years with multiple secreting-PGL/PCC tumors (including bilateral PCC with a Pheochromocytoma of the Adrenal gland Scaled Score—PASS >6 and a second primary). This case was only evaluated by a geneticist from another institution, and in the absence of MLPA studies, it should be considered as inconclusive (Table 2).

## 4 Discussion

PGL/PCC-predisposing germline variants were identified in 10 (52.63%) of 19 Colombian affected cases in our study, which is higher than expected compared to what has been reported in the literature (30–40%) (Welander et al., 2011; Fishbein, 2016; Aygun and Mehmet, 2020). Of the 12 known PPGL susceptibility genes included in the multigene cancer panel used in the Hereditary Cancer Program, only *SDHB* and *VHL* were mutated. *SDHB* is considered one of the main susceptibility genes associated with PPGL tumors (Cascón et al., 2009; Aim et al., 2021), and in this study we identified that 90% of the hereditary cases were explained by a pathogenic variant in the *SDHB* gene. Moreover, mutations in *SDHB* gene were found in the 47.37% of all the affected patients analyzed in this study. This result is similar to reports from other studies conducted in Saudi Arabia and Chinese populations in whom *SDHB* mutations were the most frequently found (Saudi Arabia: 20.8% of 101 affected cases and 57.75% of 37 inherited cases; China: 29.6% of 314 affected cases and 49.46% of 93 inherited cases) (Albattal et al., 2019); but different than other reports from European populations affected with PPGL tumors, in which *VHL* was reportedly the most frequently mutated gene (Gimenez-Roqueplo et al., 2006). Scientific evidence supports that both the prevalence and the genetic profile of hereditary PGL/PCC varies among different ethnic populations.

*SDHB* acts as a tumor suppressor gene associated as a cause of hereditary PPGL (Astuti et al., 2001). The SDHB protein (OMIM185470) corresponds to the iron-sulfur catalytic subunit of the heterotetrameric succinate dehydrogenase (SDH) complex, a component of the tricarboxylic acid cycle and the mitochondrial respiratory chain (complex II) (Aim et al., 2021). As of February 2022, 388 variants have been reported in the database of SDH mutations in the LOVD system (Bayley et al., 2005). Recent reports from an international initiative for a curated *SDHB* variant database, composed of 223 variants from 737 patients worldwide, highlights that 44% of these variants were missense, 17% were frameshift indels, 10% affects splice site, 9% were nonsense, 6% corresponded to large rearrangements and 14% to other variant types (Aim et al., 2021). This type of variant distribution persisted within the group of curated P/LP genetic variants in *SDHB*, with 34%, 26%, 15%, 14%, 9% and 3%, respectively (Aim et al., 2021). In contrast, in our study of unrelated cases, 89% (8/9) of the P/LP genetic variants in the *SDHB* gene corresponded to a large deletion of one copy of exon 1 of the gene, while the remaining 11% corresponded to a nonsense variant.

Exon 1 deletion in the *SDHB* gene has been previously reported in the literature in unrelated patients and families (McWhinney et al., 2004; Cascón et al., 2006; Cascon et al., 2007), leading to the hypothesis that this deletion may be related to a hotspot or a founder effect (Cascón et al., 2006). The molecular characterization of the deletion revealed the same breakpoints in the unrelated Spanish families studied, all originating from a small, restricted area in the northeast of the Iberian Peninsula, and that these differed from the breakpoints found in a French patient (Cascon et al., 2007). Reviewing the city of origin of the eight carriers of the recurrent CNV in the *SDHB* gene reported in this study, all but one were born in Bogotá and one patient was born in another city located 378 km southwest of Bogotá (called Neiva); nevertheless, both cities are located in the Andean region of the country. It would be interesting to test whether a founder effect similar to that observed in Spanish families is responsible for this apparent high prevalence in our Colombian patients, or if this high frequency could be explained by the presence of mutational hot spots in the *SDHB* gene. Germline haplotype information could help to better unravel the mechanisms associated with the reported recurrent mutation.

It has been reported that in carriers of P/LP variants in the *SDHB* gene, the average age at diagnosis of PPGL tumors is 31.7 years, with a range of 3–75 years; thus, the follow-up recommended in these carriers is in the childhood stage (Muth et al., 2018). In our study of Colombian cases with *SDHB* exon 1 deletion, the mean age at diagnosis of PPGL tumors (including adults and children) was 33.38 years (13–47 years), which is comparable to what has been reported in the literature for carriers of this large deletion (8–30 years) (McWhinney et al., 2004). In general, *SDHB* mutations have been associated primarily with PCC-type tumors (Ricketts et al., 2010); however, PGLs have previously been described as the primary tumor type diagnosed in cases with exon 1 deletion in the *SDHB* gene (Astuti et al., 2001; Cascón et al., 2006; Cascon et al., 2007). This is consistent with our study, since in 62% (6/8) of the patients with this large deletion in the *SDHB* gene, the initial finding was a PGL tumor. Moreover, variable expressivity was observed in relation to diverse primary PGL tumor locations and metastatic behavior, which is consistent with what has been previously reported (Ricketts et al., 2010; Else et al., 2018).

In our study, we did not observed differences regarding the age at diagnosis of metastatic disease between the group of patients with *SDHB* exon 1 deletion and the group of patients with sporadic PGL or PCC tumors, while a trend towards an earlier age at diagnosis of metastatic disease has been reported in patients carrying mutation in *SDHx* genes (especially *SDHB*) (Fishbein et al., 2017). Nevertheless, there is a discussion in the literature about a possible detection bias because in these patients a greater alert for follow-up can be generated due to the malignant potential attributed to the alteration in SDH activity (Fishbein et al., 2017; Tabebi et al., 2022). For example, it has been described that inactivating mutations in SDH complex suppress its activity, resulting in the accumulation of succinate, an oncometabolite that generates a pseudohypoxic phenotype and contributes to cancer, and that the biological effect of knocking down the *SDHB* gene is associated with a positive effect on cell proliferation, which is related to increased chromaffin cell metabolism and their adaptation to glutamine use as an alternative source of energy, thus promoting the activity of the oxidative phosphorylation system (Tabebi et al., 2022).

In the European-American-Pheochromocytoma-Paraganglioma-Registry (EAPPR), an 80% of 177 unrelated pediatric PCC/PGL patients had a germline mutation in a susceptibility gene; 38% of the patients had a second primary PGGL tumor and this trend increased with age, essentially in cases with hereditary disease, until reaching a frequency of 50% at 30 years after the initial diagnosis. Of the hereditary cases, 9% had malignant tumors and these were commonly associated with mutated *SDHB* (Bausch et al., 2014). Among the pediatric cases included in our study, Patient 17 had an initial diagnosis of left PCC at age 15 years, and second PPGL primary tumors at age 22 and 30 years managed by the oncology and endocrinology services at the INC-C, but with negative germline genetic result performed in an external laboratory. This case was evaluated by an extra-institutional medical geneticist and according to the EMR was concluded as not associated with a hereditary PGL/PCC syndrome; however, considering the high suspicion of a hereditary PGL/PPC and the elevated prevalence of exon 1 deletion in the *SDHB* gene documented in our study, it is necessary to complement the genetic studies in this patient with MLPA.

Another well-known syndrome associated with inherited PGL/PCC tumors is VHL disease, which is also associated with a variety of benign and malignant tumors, mainly retinal and cerebellar tumors, and spinal hemangioblastoma and renal cell carcinoma (van Leeuwaarde et al., 2018). This syndrome is caused by mutations in the *VHL* gene and has been reported to account for 49% of pediatric patients with PCC/PGL (Bausch et al., 2014). In our study, we only identified a *VHL* germline mutation in 1 out of 3 (33%) affected pediatric patients, corresponding to a 10% (1/10) of all of the hereditary cases.

Given the high frequency of PPGL tumors with germline mutations found in our study, we support the recommendation that genetic testing should be considered in all patients with PPGL (Plouin et al., 2016; Muth et al., 2018; Aygun and Mehmet, 2020). Equally important is to assess how complete or informative the genetic results are, by recording the type of genetic testing, the covered genes, the molecular techniques implemented, and the overall quality of the report. In our study, we used a large multigene panel that allows the analysis of 105 known cancer susceptibility genes (and candidate genes), including *MAX, NF1, RET, SDHA, SDHAF2, SDHB, SDHC, SDHD, TMEM127*, *VHL*, *FH* and *MEN1* associated with PGL/PCC predisposition. Other genes less frequently associated with inherited PGL/PCC cases (<1–2%) were not analyzed. These genes are *EGLN1, EGLN2, MDH2, SLC25A11, DLST* and *KIF1B* (Fishbein and Nathanson, 2012; Dahia, 2014; Garcia-Carbonero et al., 2021)*.* We recognized that this could be a limitation of the study, specially for the interpretation of negative cases; nevertheless, we consider that our results are comparable with other publications since these less characterized genes are not usually included in commercial multigene PGL/PCC panels.

The penetrance for *SDHB*-associated PPGL tumors has been estimated as 21% at age 70 years and 30.6% at age 80 years (Hes et al., 2010; Rijken et al., 2016; Andrews et al., 2018). In our study, a family history of PPGL was reported in 37.5% (3/8) of the patients with exon 1 deletion in the *SDHB* gene. Medical reports of genetic testing and biochemical- or imaging-surveillance screening for *SDHB*-related tumors detection among the carriers’ relatives, were not available since they were not being followed in the INC-C. We deem this to be a limitation of the study, since we could not determined the penetrance for PPGL or other related tumors.

Future studies for calculating variant penetrance using large family pedigrees and/or population data from greater cohorts are possible with the increasing accessibility of high-throughput technologies. The study design should include follow-up data obtained from cascade testing and surveillance screening. For individuals with a genetic diagnosis of hereditary PGL/PCC, cascade testing should be offered to first-degree relatives. Genetic testing for under-age relatives should be individualized according to the gene involved (Greer et al., 2017; Muth et al., 2018). Screening recommendations for presymptomatic *SDHB*-carriers include annual biochemical plasma or urine testing (comprising normetanephrine and metanephrine), accompanied by whole-body magnetic resonance imaging (RWB-MRI) every 2 years, starting 15 years. In younger children, it is recommended to design an individual surveillance program together with a pediatrician and based on family history; the recommendation to initiate surveillance 5 years before the age of the youngest affected individual in the family is generally accepted (Muth et al., 2018). Clinical examinations should be performed at least twice a year (Muth et al., 2018). Regarding follow-up after treatment in PPGL patients with a high-risk genetic disease, the European Society of Endocrinology recommends testing plasma or urinary metanephrines every year for local or metastatic recurrences or new tumors for life (Plouin et al., 2016).

It is beyond the scope of this article to comprehensively cover and discuss the complex management options for PPGL patients, which require the involvement of an expert multidisciplinary team to evaluate different factors of the individual (e.g., age, genetic profile) and the tumor (e.g., primary tumor site, hormone secretion profile, molecular profile). For interested readers, recent scientific literature about this topic can be reviewed in the following cited references (Roman-Gonzalez et al., 2018; Pryma et al., 2019; Tanabe and Naruse, 2020; Garcia-Carbonero et al., 2021).

Our findings allow us to conclude that germline mutations in *SDHB* play an important role in hereditary PGL/PCC syndromes in Colombians. Even though most of the variants reported in the *SDHx* genes (including *SDHB*) are missense mutations, the large deletion of exon 1 in *SDHB* is a recurrent mutation among our patients, explaining 42% (8/19) of the PCC and/or PGL cases, in which a variable expressivity of the phenotype associated with this large deletion was observed. The evaluation of large deletions in the *SDHB* gene should be performed in patients with PCC and/or PGL in our country.

## Data Availability

The data presented in the study are deposited in the NCBI SRA Bioproject repository (https://www.ncbi.nlm.nih.gov/sra), under the accession number PRJNA907395.

## Abbreviations

ACMG, American College of Medical Genetics and Genomics; CNV, Copy Number Variation; HGVS, Human Genome Variation Society; INDEL, Insertion-Deletion Mutation; INC-C, Instituto Nacional de Cancerología, Colombia; 131I MIBG, 131I-meta-iodobenzylguanidine; MEN2, Multiple Endocrine Neoplasia Type 2; MLPA, Multiplex Ligation-Dependent Probe Amplification; N/A, Not Applicable; NGS, Next Generation Sequencing; P/LP, Pathogenic or Likely Pathogenic; PCC, Pheochromocytoma; PGL, Paraganglioma; PPGL, Pheochromocytomas and Paragangliomas in combination; PRRT, Peptide Receptor Radionuclide Therapy; RWB-MRI, Whole-Body Magnetic Resonance Imaging; SDHB, Succinate Dehydrogenase Subunit B; SDHx, Genes encoding subunits of Succinate Dehydrogenase Enzyme; SNV, Single Nucleotide Variant; VHL, Von Hippel-Lindau; VUS, Variant of Uncertain Significance.
